# Safety and immunogenicity of two novel type 2 oral poliovirus vaccine candidates compared with a monovalent type 2 oral poliovirus vaccine in healthy adults: two clinical trials

**DOI:** 10.1016/S0140-6736(20)32541-1

**Published:** 2021-01-02

**Authors:** Ilse De Coster, Isabel Leroux-Roels, Ananda S Bandyopadhyay, Christopher Gast, Kanchanamala Withanage, Katie Steenackers, Philippe De Smedt, Annelies Aerssens, Geert Leroux-Roels, M Steven Oberste, Jennifer L Konopka-Anstadt, William C Weldon, Alan Fix, John Konz, Rahnuma Wahid, John Modlin, Ralf Clemens, Sue Ann Costa Clemens, Novilia S Bachtiar, Pierre Van Damme

**Affiliations:** a Centre for the Evaluation of Vaccination, Vaccine and Infectious Disease Institute, University of Antwerp, Wilrijk, Belgium; b Center for Vaccinology, Ghent University and Ghent University Hospital, Ghent, Belgium; c Bill & Melinda Gates Foundation, Seattle, WA, USA; d PATH, Washington DC, USA; e Division of Viral Diseases, National Center for Immunization and Respiratory Diseases, Centers for Disease Control and Prevention, Atlanta, GA, USA; f Dartmouth Geisel School of Medicine, Hanover, NH, USA; g Global Research in Infectious Diseases, Rio de Janeiro, Brazil; h Institute for Global Health, Siena University, Siena, Italy; i PT Bio Farma, Bandung, Indonesia

## Abstract

**Background:**

Two novel type 2 oral poliovirus vaccine (OPV2) candidates, novel OPV2-c1 and novel OPV2-c2, designed to be more genetically stable than the licensed Sabin monovalent OPV2, have been developed to respond to ongoing polio outbreaks due to circulating vaccine-derived type 2 polioviruses.

**Methods:**

We did two randomised studies at two centres in Belgium. The first was a phase 4 historical control study of monovalent OPV2 in Antwerp, done before global withdrawal of OPV2, and the second was a phase 2 study in Antwerp and Ghent with novel OPV2-c1 and novel OPV2-c2. Eligible participants were healthy adults aged 18–50 years with documented history of at least three polio vaccinations, including OPV in the phase 4 study and either OPV or inactivated poliovirus vaccine (IPV) in the novel OPV2 phase 2 study, with no dose within 12 months of study start. In the historical control trial, participants were randomly assigned to either one dose or two doses of monovalent OPV2. In the novel OPV2 trial, participants with previous OPV vaccinations were randomly assigned to either one or two doses of novel OPV2-c1 or to one or two doses of novel OPV2-c2. IPV-vaccinated participants were randomly assigned to receive two doses of either novel OPV2-c1, novel OPV2-c2, or placebo. Vaccine administrators were unmasked to treatment; medical staff performing safety and reactogenicity assessments or blood draws for immunogenicity assessments were masked. Participants received the first vaccine dose on day 0, and a second dose on day 28 if assigned to receive a second dose. Primary objectives were assessments and comparisons of safety up to 28 days after each dose, including solicited adverse events and serious adverse events, and immunogenicity (seroprotection rates on day 28 after the first vaccine dose) between monovalent OPV2 and the two novel OPV2 candidates. Primary immunogenicity analyses were done in the per-protocol population. Safety was assessed in the total vaccinated population—ie, all participants who received at least one dose of their assigned vaccine. The phase 4 control study is registered with EudraCT (2015-003325-33) and the phase 2 novel OPV2 study is registered with EudraCT (2018-001684-22) and ClinicalTrials.gov (NCT04544787).

**Findings:**

In the historical control study, between Jan 25 and March 18, 2016, 100 volunteers were enrolled and randomly assigned to receive one or two doses of monovalent OPV2 (n=50 in each group). In the novel OPV2 study, between Oct 15, 2018, and Feb 27, 2019, 200 previously OPV-vaccinated volunteers were assigned to the four groups to receive one or two doses of novel OPV2-c1 or novel OPV2-c2 (n=50 per group); a further 50 participants, previously vaccinated with IPV, were assigned to novel OPV2-c1 (n=17), novel OPV2-c2 (n=16), or placebo (n=17). All participants received the first dose of assigned vaccine or placebo and were included in the total vaccinated population. All vaccines appeared safe; no definitely vaccine-related withdrawals or serious adverse events were reported. After first doses in previously OPV-vaccinated participants, 62 (62%) of 100 monovalent OPV2 recipients, 71 (71%) of 100 recipients of novel OPV2-c1, and 74 (74%) of 100 recipients of novel OPV2-c2 reported solicited systemic adverse events, four (monovalent OPV2), three (novel OPV2-c1), and two (novel OPV2-c2) of which were considered severe. In IPV-vaccinated participants, solicited adverse events occurred in 16 (94%) of 17 who received novel OPV2-c1 (including one severe) and 13 (81%) of 16 who received novel OPV2-c2 (including one severe), compared with 15 (88%) of 17 placebo recipients (including two severe). In previously OPV-vaccinated participants, 286 (97%) of 296 were seropositive at baseline; after one dose, 100% of novel OPV2 vaccinees and 97 (97%) of monovalent OPV2 vaccinees were seropositive.

**Interpretation:**

Novel OPV2 candidates were as safe, well tolerated, and immunogenic as monovalent OPV2 in previously OPV-vaccinated and IPV-vaccinated adults. These data supported the further assessment of the vaccine candidates in children and infants.

**Funding:**

University of Antwerp and Bill & Melinda Gates Foundation.

**Research in context**

**Evidence before this study**

Despite the global eradication of wild-type 2 poliovirus, and the global withdrawal of live-type 2 poliovirus from oral poliovirus vaccines (OPVs), there are increasing cases of type 2 poliomyelitis outbreaks due to type 2 circulating vaccine-derived polioviruses. As members of the consortium involved in this unique development of new OPV vaccines, we did not perform a literature search. We published the only previous clinical study assessing one dose of clinical trial lots of novel attenuated Sabin strain poliovirus vaccine candidates (novel OPV2) designed to be more genetically stable than Sabin monovalent OPV2. In that study, one dose of each candidate was administered to two small groups of adult volunteers (n=15 each) living in containment for 28 days to assess the safety, tolerability, immunogenicity, and stability of attenuation after intestinal passage.

**Added value of this study**

This study is the first to show that in an adult population with a history of previous OPV vaccination, both novel OPV2 vaccine candidates are safe and well tolerated and have statistical non-inferiority for immunogenicity compared with the monovalent OPV2 vaccine they are designed to replace. This study is also the first to elicit evidence of induction of primary intestinal immunogenicity with novel OPV2 in adults with no previous exposure to live type 2 poliovirus (exclusively inactivated polio vaccine vaccinated) in whom rates of shedding were lower after a second dose. Furthermore, preliminary assessments have confirmed the higher genetic stability of shed vaccine virus. Further investigation of these exploratory findings is ongoing to confirm the nature of any detectable changes in shed virus and to assess potential neurovirulence of shed virus in comparison with licensed monovalent OPV2.

**Implications of all the available evidence**

The rapid increase in cases of type 2 circulating vaccine-derived polioviruses was designated a Public Health Emergency of International Concern by WHO in 2014 and the Global Polio Eradication Initiative response has been included in a 2020–21 addendum to the Polio Endgame Strategy. Continued use of Sabin monovalent OPV2 to interrupt the ongoing outbreaks in settings of poor immunisation coverage risks seeding further type 2 circulating vaccine-derived polioviruses because of its inherent tendency to lose the attenuating mutations. This unique epidemiological situation necessitates an urgent need for development and introduction of more genetically stable type 2 polioviruses (novel OPV2) to respond to the global emergency. The data from this study in adults confirms that two novel OPV2 candidates are safe, well tolerated, and have immunogenicity similar to Sabin monovalent OPV2 and improved genetic stability after intestinal passage. These data enabled the initiation of the studies with novel OPV2 in children and infants and the subsequent application for review under the Emergency Use Listing procedure for early use of novel OPV2 in outbreak control.

## Introduction

Global eradication of wild-type 2 and 3 polioviruses has been declared,^1^ with wild-type 1 now only endemic in Afghanistan and Pakistan.^2^ However, intestinal reversion to neurovirulence of attenuated Sabin oral poliovirus vaccine (OPV) viruses can occur and, when shed in stools and transmitted through populations with low OPV coverage, it can cause cases of paralysis.^3^ Reported numbers of such circulating vaccine-derived poliovirus cases have increased every year since 2016, mainly due to type 2.^4^ The Global Polio Eradication Initiative has developed a response strategy, which includes the development of new vaccines.^5^

A consortium has been working since 2011 on research and development of novel poliovirus strains engineered to be more genetically stable with less likelihood of reversion to neurovirulence while retaining the benefits of Sabin OPV. Because more than 94% of circulating vaccine-derived poliovirus cases were due to type 2, initial focus was on novel type 2 OPVs (OPV2s),^6^ and has produced two candidates, OPV2-c1 and OPV2-c2.^7^, ^8^ Both candidates are attenuated serotype 2 polioviruses derived from a modified Sabin 2 infectious clone with different combinations of five distinct modifications of the Sabin 2 genome, propagated in Vero cells. Novel OPV2-c1 includes a genetically stabilised domain V (the primary attenuation site for Sabin 2), relocation of the cis-acting replication element, and modifications to the polymerase to enhance fidelity and reduce recombination.^7^ Novel OPV2-c2 includes the same genetically stabilised domain V and codon deoptimisation in the capsid-coding region.^8^ These modifications aimed to stabilise the genetic sequence against reversion in the 5′ untranslated region with additional attenuation provided by introducing about 87 additional silent mutations in the capsid region.

After reporting the first phase 1 study of both candidates in healthy adults,^9^ we now report a larger phase 2 assessment of the safety, tolerability, immunogenicity, and genetic stability of both candidates in adults vaccinated with OPV or inactivated polio vaccine (IPV) to support further clinical development in children and infants.^10^ This investigation is unique because global withdrawal of type 2-containing OPVs during the development of the novel OPV2 vaccines before clinical trial lots were available made it impossible to concurrently compare monovalent OPV2 and novel OPV2. Therefore, we did a prospectively designed phase 4 study with monovalent OPV2 vaccine to provide historical control data against which to assess each novel OPV2 candidate. Both studies are reported here.

## Methods

### Study design and participants

We did two partially masked studies at two centres: a phase 4 study of monovalent OPV2 (historical control study) at the Centre for the Evaluation of Vaccination, Vaccine and Infectious Disease Institute, University of Antwerp (Antwerp, Belgium); and a phase 2 study of the two novel OPV2 candidates at the same centre and at the CEVAC, Center for Vaccinology, Ghent University Hospital (Ghent, Belgium). Study protocols were approved by each centre's institutional review board and the Belgian national authority. The studies were conducted according to the Declaration of Helsinki and International Conference on Harmonisation Good Clinical Practice guidelines. All participants provided written informed consent.

Eligible participants were healthy adults aged 18–50 years with documented history of at least three polio vaccinations, including OPV in the phase 4 study and either OPV or IPV in the novel OPV2 phase 2 study, with no dose within 12 months of study start. Other inclusion criteria were being a resident in Belgium and being available for the study duration, and being in good mental and physical health at enrolment on the basis of medical history and examination. Females of childbearing potential had to have a negative pregnancy test at enrolment and agree to use an approved contraceptive method during and for 3 months after the study. Main exclusion criteria were any medical condition likely to affect the participant's wellbeing or immune response, including a low baseline total serum IgA level, any travel intended or within the previous 6 months to polio-endemic countries, breastfeeding, any professional food handling duties, any professional or household contact with immunosuppressed or incompletely polio-vaccinated people (eg, young infants), or participation in another clinical trial within 28 days of this one.

### Randomisation and masking

Historical control study participants were enrolled and randomly assigned 1:1 to receive one or two doses of monovalent OPV2. In the novel OPV2 study, the novel OPV2-c2 candidate was prioritised so the first 100 OPV-vaccinated participants were randomly assigned 1:1 to groups 3 (one dose) and 4 (two doses) to receive novel OPV2-c2. The second 100 OPV-vaccinated participants were randomly assigned 1:1 to groups 1 (one dose) and 2 (two doses) to receive novel OPV2-c1. IPV-vaccinated adults were enrolled in parallel and randomly assigned 2:1 to group 6 (two doses of novel OPV2-c2) or group 7 (two doses of placebo), until group 6 enrolment was complete, when 2:1 randomisation was continued for group 5 (two doses of novel OPV2-c1) and group 7 (two doses of placebo). Block randomisation was used throughout to ensure balanced randomisation across time using a preprepared computer-generated randomisation schedule (Assign Data Management and Biostatistics, Innsbruck, Austria). The study nurses (administration team) who gave the vaccine or placebo were unmasked according the randomisation schedule, but each participant and the medical staff who assessed adverse events and drew blood samples for immunogenicity assessments were masked as to vaccine to placebo assignment.

### Procedures

The monovalent OPV2 vaccine was Polio Sabin Mono Two (oral), manufactured by GlaxoSmithKline Biologicals, Belgium; lot number mOPV2-007, batch number DOP2A004AZ. The vaccine is a licensed, monovalent, live-attenuated poliomyelitis virus vaccine of the Sabin strain type 2 (P 712, Ch, 2ab), propagated in MRC5 human diploid cells. Each two-drop dose (0·1 mL) nominally contained 10^5·7^ 50% cell culture infective dose (CCID_50_) units of type 2 poliovirus at release.

Both novel OPV2 candidates, novel OPV2-c1 (lot number 2060416C) and novel OPV2-c2 (lot number 2060316C), were manufactured by Bio Farma (Jawa Barat, Indonesia). High doses of novel OPV2 containing about 1 000 000 CCID_50_ to ensure robust safety assessments, were administered orally as six drops (0·3 mL) delivered from a supplied dropper. Placebo was six orally administered drops of sugar syrup, propylene glycol (batch number 18B06/V89669; Conforma, Destelbergen, Belgium). One-dose groups received their only dose on day 0; two-dose groups received one dose on day 0 and the second on day 28.

Participants were monitored for 30 min after vaccination for immediate reactions, then asked to complete 7-day diary cards soliciting systemic adverse events and daily oral temperature, which were graded for severity as follows: mild (easily tolerated with minimal discomfort, 37·5–38·0°C), moderate (sufficiently discomforting to interfere with normal everyday activities, 38·1–39·0°C) or severe (prevents normal everyday activities, >39·0°C). Unsolicited adverse events were recorded for 28 days after each vaccination and assessed for causality and severity by the study investigator. Terms used to identify adverse events were coded according to the Medical Dictionary for Regulatory Activities (version 22.0). A standard panel of clinical laboratory assessments in the historical study was augmented with measurements of creatine phosphokinase, γ-glutamyl transferase, and albumin in the novel OPV2 study after observation of increased levels of creatine phosphokinase and some liver enzymes in some participants in the phase 1 study of both novel OPV2 candidates.^9^

Sera obtained on days 0, 28, and 56 (after two doses) were stored and shipped at a maximum temperature of –20°C to the Centers for Disease Control and Prevention (CDC) laboratories (Atlanta, GA, USA) for measurement of poliovirus type 2-specific antibodies concurrently for both studies using the WHO standard microneutralisation assay (WHO EPI GEN 93.9), adapted as previously described.^9^, ^11^ The lower limit of quantitation (LLOQ) was 2·5 log_2_ titre and the upper limit of quantitation (ULOQ) was 10·5 log_2_ titre. At each timepoint we calculated seroprotection rates (group proportions with a neutralising antibody titre ≥1:8), group geometric mean titres using a logarithmic (base 2) scale, and seroconversion rates (total proportions of each group who changed from seronegative to seropositive or, for those who were initially seropositive, who displayed an at least four-fold rise in antibody titres after vaccination). Seroconversion was only calculated in individuals whose baseline antibody titre was low enough to allow observation of a four-fold increase without breaching the ULOQ.

Daily stool samples collected at days 0–10, 14, 21, 28, and 42 in one-dose groups, and additionally at days 29–38, 42, 49, 56 and 70 in two-dose groups, were stored and shipped to the CDC laboratory as were the serum samples. Nucleic acid was extracted from stool samples to detect poliovirus using RT-PCR and, in positive samples, the viral load was measured as CCID_50_.^12^ Deep-sequencing was done in exploratory endpoint stool samples, representing each participant's last polio type 2-positive stool samples containing more than 4·00 log_10_ CCID_50_ per g of stool, using cDNA synthesis and full-length poliovirus genome amplification as described previously.^9^

### Outcomes

Coprimary objectives were to assess and compare safety of novel OPV2 versus monovalent OPV2 in OPV-vaccinated groups, or novel OPV2 versus placebo in IPV-vaccinated groups, in terms of serious and severe adverse events up to day 28 after the first dose of vaccine, and immunogenicity as seroprotection rate 28 days after one dose in OPV-vaccinated groups. Secondary objectives were assessments of systemic reactogenicity, assessed as solicited adverse events for 7 days after each vaccination and as unsolicited adverse events for 28 days after each vaccination; and immunogenicity. Immunogenicity parameters included geometric mean titres of poliovirus neutralising antibodies at all measured timepoints, seroprotection rates at timepoints other than day 28 (primary objective), and seroconversion rates. Exploratory objectives were measurements of viral shedding and the genetic stability of any shed virus in stool viral samples. Ultimately, samples of shed virus will be assessed for neurovirulence, but this is beyond the scope of this report.

### Statistical analysis

Sample size for OPV-vaccinated groups for each study was selected considering a non-inferiority comparison of seroprotection rates between each candidate and the control after one vaccination, assuming a 95% seroprotection rate, one-sided α=0·025, margin 10%, and 80% power, and augmented to ensure at least 50 participants were allocated to each dose group to achieve a 90% probability of observing an adverse event of interest when the true rate was 5%, allowing for a 5% dropout. Sample sizes for IPV-vaccinated groups were selected to detect a four-times increase in the risk of specific increased laboratory values assuming a background rate of 6%, using one-sided α=0·05 and 80% power, and allowing for 5% dropout.

All adverse events, including serious adverse events, severe adverse events, and solicited and unsolicited adverse events were summarised by type, seriousness, severity, and causality and by group and overall, and primary safety endpoints were compared between corresponding monovalent OPV2 (groups 1 and 2) and novel OPV2 (groups 1–4) and between novel OPV2 and placebo for exclusively IPV-vaccinated participants (groups 5 and 6 *vs* group 7) using the two-sided Fisher's exact test after each dose individually, and across all doses. The primary immunogenicity endpoint, the seroprotection rate after one dose of either vaccine candidate in the OPV-vaccinated groups (novel OPV2 study combined groups 1 and 2, and combined groups 3 and 4), was compared with the corresponding endpoint from the historical monovalent OPV2 control study (combined groups 1 and 2) via a non-inferiority test of the difference of each of the novel candidates to the monovalent OPV2 control, each using one-sided α=0·025 and a non-inferiority margin of 10%, computed using two-sided α=0·05 Miettinen and Nurminen score-based CIs for inference. The method used was described previously.^13^ The independent variables are the vaccine group indicator and the baseline titre; the dependent variable is the post-baseline titre, which is considered to be observed, right censored (if result is ≥ULOQ), or left censored (if result is ≤LLOQ), to avoid bias in estimation due to the expected high frequency of responses exceeding ULOQ because of previous vaccinations received.

Secondary endpoints for OPV-vaccinated participants involved similar comparisons between corresponding groups across studies (monovalent OPV2 study groups 1 and 2 compared with novel OPV2 study groups 1 and 2, and groups 3 and 4) using two-sided 95% CIs for the rate difference (seroconversion rate), the difference in medians (log_2_ neutralising titres, using bootstrap methods), or the neutralising antibody geometric mean titre ratio, using survival regression analysis on the log_2_ titres with normal errors, incorporating the baseline log_2_ titres as a covariate, and using maximum likelihood estimation to accommodate censoring at ULOQ and LLOQ, with reverse transformation of the model-estimated difference in means and corresponding CI. Immunogenicity data from IPV-vaccinated participants (monovalent OPV2 study groups 5–7) were summarised with the seroprotection rates, seroconversion rates, and geometric mean titres, but not compared between groups.

For each timepoint, viral shedding positivity and concentration were summarised. A viral shedding index estimate calculated for each participant as the average of log_10_-transformed values of CCID_50_ per g in stool samples established using quantitative PCR (viral identity) and CCID_50_ (titres) from select stool samples taken 7, 14, 21, and 28 days after each vaccination was summarised by group and dose. Assay LLOQ (2·75 log_10_ CCID_50_ per g) and ULOQ (8·25 log_10_ CCID_50_ per g) were used as observed values where necessary.

Primary immunogenicity analyses were done in the per-protocol population. Safety was assessed in the total vaccinated population—ie, all participants who received at least one dose of their assigned vaccine.

An independent data and safety monitoring board monitored the novel OPV2 development programme, including the previous phase 1 study,^9^ the present novel OPV2 study, and another in children and infants.^10^ The phase 4 control study is registered with EudraCT (2015-003325-33) and the phase 2 novel OPV2 study is registered with EudraCT (2018-001684-22) and ClinicalTrials.gov (NCT04544787).

### Role of the funding source

The funder was involved in study design, data analysis, data interpretation, and writing of the report, but had no role in data collection. All authors had full access to all the data in the study and had final responsibility for the decision to submit for publication.

## Results

In the historical control study, between Jan 25 and March 18, 2016, 112 volunteers were screened and 100 were enrolled and assigned to receive one or two doses of monovalent OPV2. All 100 participants received the assigned number of doses and remained in the study to the end of follow-up (day 42 for those in the one-dose group and day 70 for those in the two-dose group; figure A). In the novel OPV2 study, between Oct 15, 2018, and Feb 27, 2019, 277 volunteers were screened and 250 were enrolled (200 OPV vaccinated and 50 IPV vaccinated). Enrolment of IPV-vaccinated participants was truncated, per protocol, because of low enrolment rates, with data and safety monitoring board concurrence on the accumulation of sufficient safety data in these groups. Of the OPV-vaccinated participants, 50 were assigned to each of the four groups and of the 50 IPV-vaccinated participants, 17 were assigned to novel OPV2-c1, 16 to novel OPV2-c2, and 17 to placebo (figure B).

**Figure:**
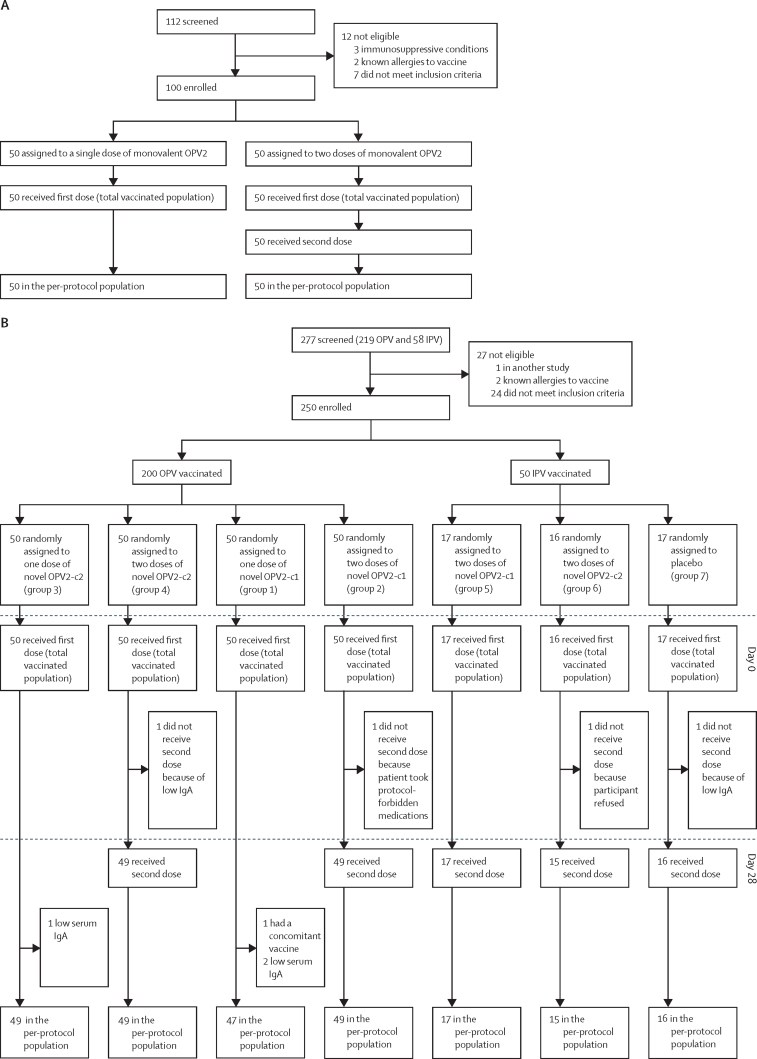
Trial profiles for the historical study with monovalent OPV2 (A) and the new study with novel OPV2 candidates (B) c1=candidate 1. c2=candidate 2. IPV=inactivated poliovirus vaccine. OPV=oral poliovirus vaccine. OPV2=type 2 OPV.

All participants received at least one vaccination and were included in the total vaccinated population for analysis of safety. Eight participants were excluded from the per-protocol population for immunogenicity analyses, either because they had low IgA, did not receive their assigned second vaccinations, or received concomitant medication not permitted by the protocol.

Demographics were generally similar across studies and groups in terms of age, race, and body-mass index, except for the male to female ratio (table 1). In OPV-vaccinated groups across both studies, 133 (44%) of 300 were men and 167 (56%) were women, and in the IPV-vaccinated groups, 12 (24%) of 50 were men and 38 (76%) were women. Most OPV-vaccinated participants had received three or four vaccinations and most IPV-vaccinated participants had received four to six vaccinations.

**Table 1:** Demographics of the populations in the two studies (all vaccinated participants)

			**Historical control study**		**Novel OPV2 study—OPV vaccinated**				**Novel OPV2 study—IPV vaccinated**		
			Monovalent OPV2, group 1 (n=50)	Monovalent OPV2, group 2 (n=50)	Novel OPV2-c1, group 1 (n=50)	Novel OPV2-c1, group 2 (n=50)	Novel OPV2-c2, group 3 (n=50)	Novel OPV2-c2, group 4 (n=50)	Novel OPV2-c1, group 5 (n=17)	Novel OPV2-c2, group 6 (n=16)	Placebo, group 7 (n=17)
Age, years			26 (8)	28 (9)	31 (10)	31 (10)	32 (10)	34 (10)	23 (10)	31 (9)	24 (8)
Sex											
	Female		31 (62%)	25 (50%)	22 (44%)	29 (58%)	28 (56%)	32 (64%)	11 (65%)	13 (81%)	14 (82%)
	Male		19 (38%)	25 (50%)	28 (56%)	21 (42%)	22 (44%)	18 (36%)	6 (35%)	3 (19%)	3 (18%)
Body-mass index, kg/m^2^			22·8 (3·3)	24·7 (4·5)	24·2 (4·2)	23·8 (3·3)	25·0 (4·4)	25·3 (3·6)	23·1 (4·8)	23·9 (4·7)	24·7 (4·2)
Race											
	White		48 (96%)	49 (98%)	49 (98%)	49 (98%)	49 (98%)	49 (98%)	16 (94%)	16 (100%)	15 (88%)
	Asian		2 (4%)	0	0	0	1 (2%)	0	0	0	0
	Black or African American		0	1 (2%)	0	0	0	0	0	0	1 (6%)
	Other		0	0	1 (2%)	1 (2%)	0	1 (2%)	1 (6%)	0	1 (6%)
Documented polio vaccination history*											
	OPV doses										
		3	6 (12%)	6 (12%)	49 (98%)	50 (100%)	41 (82%)	40 (80%)	..	..	..
		4	44 (88%)	44 (88%)	1 (2%)	0	9 (18%)	9 (18%)	..	..	..
		5	0	0	0	0	0	1 (2%)	..	..	..
	IPV doses										
		0	46 (92%)	47 (94%)	46 (92%)	49 (98%)	48 (96%)	47 (94%)	0	0	0
		1	4 (8%)	3 (6%)	4 (8%)	1 (2%)	2 (4%)	3 (6%)	0	0	0
		4	..	..	..	..	..	..	6 (35%)	4 (25%)	10 (59%)
		5	..	..	..	..	..	..	10 (59%)	5 (31%)	5 (29%)
		6 or more	..	..	..	..	..	..	1 (6%)	7 (44%)	2 (12%)

Data are mean (SD) or n (%). c1=candidate 1. c2=candidate 2. IPV=inactivated poliovirus vaccine. OPV=oral poliovirus vaccine. OPV2=type 2 OPV.

* All participants were assumed to have at least three doses as required by the Belgian legislation if they were unable to produce vaccination cards; OPV-vaccinated participants could have also received IPV, but IPV participants were specifically only to have received IPV, no OPV dose.

No deaths, life-threatening conditions, or definitely related serious adverse events were reported, no participant withdrew from either study because of adverse events, and no differences in proportions of patients with primary safety endpoint events were observed, except for a higher rate of any severe unsolicited events after first dose (one [6%] of 16 with novel OPV2-c2 *vs* seven [41%] of 17 with placebo) in IPV-vaccinated participants (table 2). Of the four serious adverse events, all in the novel OPV2 study, one was possibly related to vaccination; an IPV-vaccinated (group 6) participant had an influenza-like illness with onset 12 days after a second dose of novel OPV2-c2 that lasted for 6 days before resolving. Three other serious adverse events were considered unrelated to vaccination; a new onset ileitis terminalis (group 1, novel OPV2-c1) diagnosed 56 days after vaccination, and cases of severe vomiting (group 2, novel OPV2-c1) due to medication for shoulder surgery and anaphylaxis (group 4, novel OPV2-c2) due to medication for cystitis.

**Table 2:** Participants reporting solicited adverse events within 7 days of vaccination, and unsolicited adverse events within 28 days, of each vaccination in all vaccinated participants

		**Historical control study**	**Novel OPV2 study—OPV vaccinated**		**Novel OPV2 study—IPV vaccinated**		
		Monovalent OPV2, groups 1 and 2	Novel OPV2-c1, groups 1 and 2	Novel OPV2-c2, groups 3 and 4	Novel OPV2-c1, group 5	Novel OPV2-c2, group 6	Placebo, group 7
**Solicited systemic adverse events after dose 1**							
N		100	100	100	17	16	17
	Any	62 (62%)	71 (71%)	74 (74%)	16 (94%)	13 (81%)	15 (88%)
	Mild	47 (47%)	45 (45%)	60 (60%)	15 (88%)	12 (75%)	14 (82%)
	Moderate	11 (11%)	23 (23%)	12 (12%)	4 (24%)	7 (44%)	5 (29%)
	Severe	4 (4%)	3 (3%)	2 (2%)	1 (6%)	0	2 (12%)
**Solicited systemic adverse events after dose 2**							
N*		50	49	49	17	15	16
	Any	18 (36%)	26 (53%)	21 (43%)	11 (65%)	9 (60%)	12 (75%)
	Mild	10 (20%)	18 (37%)	14 (29%)	8 (47%)	3 (20%)	7 (44%)
	Moderate	7 (14%)	8 (16%)	6 (12%)	3 (18%)	5 (33%)	5 (31%)
	Severe	1 (2%)	0	1 (2%)	0	1 (7%)	0
**Unsolicited adverse events after dose 1**							
N		100	100	100	17	16	17
	Any	65 (65%)	68 (68%)	69 (69%)	13 (76%)	12 (75%)	16 (94%)
	Probably or possibly related	28 (28%)	19 (19%)	19 (19%)	1 (6%)	2 (13%)	1 (6%)
	Severe	13 (13%)	18 (18%)	7 (7%)	3 (18%)	1 (6%)	7 (41%)
**Unsolicited adverse events after dose 2**							
N*		50	49	49	17	15	16
	Any	26 (52%)	33 (67%)	33 (67%)	11 (65%)	12 (80%)	14 (88%)
	Probably or possibly related	7 (7%)	5 (10%)	7 (14%)	1 (6%)	1 (7%)	0
	Severe	5 (10%)	6 (12%)	5 (10%)	1 (6%)	4 (27%)	5 (31%)

Data are n (%) unless otherwise stated. c1=candidate 1. c2=candidate 2. IPV=inactivated poliovirus vaccine. OPV=oral poliovirus vaccine. OPV2=type 2 OPV.

* Only includes two-dose groups.

There were no meaningful differences in reactogenicity between the monovalent OPV2 and novel OPV2 groups. Most OPV-vaccinated participants reported solicited adverse events within 7 days of their first vaccination, 62 (62%) of 100 after monovalent OPV2, 71 (71%) of 100 after novel OPV2-c1, and 74 (74%) of 100 after novel OPV2-c2 (table 2). Most frequent adverse events were headache, fatigue, abdominal pain, diarrhoea, and myalgia, with no difference in frequency or severity across groups (appendix p 2). Most were mild to moderate, but across both studies, nine OPV-vaccinated participants reported severe adverse events, including cases of headache (six participants), myalgia (two participants), and fatigue and paraesthesia (one participant each), all of which resolved. Rates of solicited adverse events in OPV-vaccinated groups were lower after the second dose than after the first dose, reported by 18 (36%) of 50 after monovalent OPV2, 26 (53%) of 49 after novel OPV2-c1, and 21 (43%) of 49 participants after novel OPV2-c2 (appendix p 3). One case of abdominal pain and one of fatigue were described as severe.

In the IPV-vaccinated groups, solicited adverse event rates were higher, with 16 (94%) of 17 in the novel OPV2-c1 group, 13 (81%) of 16 in the novel OPV2-c2 group, and 15 (88%) of 17 in the placebo group (table 2). Four participants reported solicited severe adverse events (appendix pp 2–3): three after first doses of either placebo (one with headache and fatigue and one with fatigue) or novel OPV-c1 (one with severe headache) and one after the second dose of novel OPV-c2 (severe fatigue).

Most participants reported an unsolicited adverse event during the study (table 2), with severe unsolicited adverse events reported by 17 (17%) of 100 monovalent OPV2 recipients in the historical study, compared with 23 (23%) of 100 after novel OPV2-c1 and 11 (11%) of 100 after novel OPV2-c2 in the OPV-vaccinated groups. In IPV-vaccinated participants, four (24%) of 17 after novel OPV2-c1 and five (31%) of 16 after novel OPV2-c2 reported severe unsolicited adverse events, compared with nine (53%) of 17 placebo recipients. Relationship to vaccination was considered to be possible or probable for four severe unsolicited adverse events after monovalent OPV2, and for two after novel OPV2-c1 and four after novel OPV2-c2 in OPV-vaccinated and IPV-vaccinated groups, and for three participants who received placebo. These severe adverse events mainly consisted of gastrointestinal disorders—diarrhoea, nausea, and abdominal pain occurring after the 7-day solicited adverse event reporting period.

There were no consistent abnormalities in clinical laboratory assessments related to receipt of either novel OPV2-c1 or novel OPV2-c2 in OPV-vaccinated or IPV-vaccinated participants (appendix p 4). Four clinically relevant grade 4 laboratory abnormalities were observed; three increases of creatine kinase—two in OPV-vaccinated participants at day 28 after the first dose of novel OPV2-c2 (which were linked to practising sport) and one in an IPV-vaccinated participant 7 days after placebo—and a grade 4 potassium level increase observed at day 56 after two doses on monovalent OPV2 in the historical study, possibly due to haemolysis. Overall, frequencies of grade 3 or 4 outcomes were no greater after vaccination than at baseline (day 0). Furthermore, no grade 3 or 4 changes in alanine aminotransferase or aspartate aminotransferase, or the related parameters γ-glutamyl transferase, bilirubin, or albumin, were observed.

At baseline, 286 (97%) of 296 OPV-vaccinated participants across both studies—(97 [97%] of 100 in the monovalent OPV2 study, 189 [96%] of 196 in the novel OPV2 study)—were already seropositive for poliovirus type 2 (table 3), precluding any meaningful comparisons between vaccine groups. Overall, immune responses to the novel OPV2 candidates as seroprotection rate, median titres, or geometric mean titres appeared to be similar to or greater than those observed after monovalent OPV2 in the historical control study (appendix p 5).

**Table 3:** Median poliovirus neutralising antibody titres and seroprotection and seroconversion rates in the per-protocol population

		**Historical control study**	**Novel OPV2 study—OPV vaccinated**		**Novel OPV2 study—IPV vaccinated**		
		Monovalent OPV2, groups 1 and 2	Novel OPV2-c1, groups 1 and 2	Novel OPV2-c2, groups 3 and 4	Novel OPV2-c1, group 5	Novel OPV2-c2, group 6	Placebo, group 7
**Poliovirus neutralising antibody titres**							
Day 0, baseline							
	N	100	98	98	15	16	16
	Median (95% CI), log_2_	7·83 (7·34–8·50)	8·34 (7·83–8·83)	8·83 (8·00–9·50)	7·83 (6·50–9·17)	7·17 (4·83–8·50)	6·50 (3·83–8·00)
Day 28, after dose 1							
	N	100	96	98	17	16	16
	Median (95% CI), log_2_	9·67 (8·34–10·17)	10·50 (10·50–10·50)	10·17 (9·67–10·5)	10·50 (10·50–10·50)	10·50 (10·17–10·50)	5·67 (3·50–7·83)
Day 56, after dose 2*							
	N	50	49	49	17	15	16
	Median (95% CI), log_2_	10·17 (8·50–10·50)	10·50 (10·50–10·50)	10·50 (9·50–10·50)	10·50 (10·50–10·50)	10·50 (9·17–10·5)	7·00 (4·50–8·50)
**Seroprotection rates**							
Day 0, baseline							
	N	100	98	98	15	16	16
	n (%; 95% CI)	97 (97%; 92–99)	97 (99%; 94–100)	92 (94%; 87–98)	14 (93%; 68–100)	15 (94%; 70–100)	13 (81%; 54–96)
Day 28, after dose 1							
	N	100	96	98	17	16	16
	n (%; 95% CI)	97 (97%; 92–99)	96 (100%; 96–100)	98 (100%; 96–100)	17 (100%; 81–100)	16 (100%; 79–100)	12 (75%; 48–93)
Day 56, after dose 2*							
	N	50	49	49	17	15	16
	n (%; 95% CI)	49 (98%; 89–100)	49 (100%; 93–100)	49 (100%; 93–100)	17 (100%; 81–100)	15 (100%; 78–100)	13 (81%; 54–96)
**Seroconversion rates**†							
Day 28, after dose 1							
	N	62	55	47	10	12	12
	n (%; 95% CI)	18 (29%; 18–42)	41 (75%; 61–83)	24 (51%; 36–66)	10 (100%; 69–100)	11 (92%; 62–100)	0 (0%; 0–26)
Day 56, after dose 2*							
	N	29	27	26	10	11	12
	n (%; 95% CI)	11 (38%; 21–58)	20 (74%; 54–89)	15 (58%; 37–77)	10 (100%; 69–100)	9 (82%; 48–98)	1 (8%; 0–38)

Log_2_ titre values shown as 2·5 should be interpreted as 2·50 or less and the use of 10·50 should be interpreted as 10·50 or greater. c1=candidate 1. c2=candidate 2. IPV=inactivated poliovirus vaccine. OPV=oral poliovirus vaccine. OPV2=type 2 OPV.

* Only includes two-dose groups.

† Seroconversion was only measured in those whose initial antibody titre allowed observation of a four-fold increase.

Monovalent OPV2 in 100 OPV-vaccinated participants increased the median log_2_ titre from 7·83 (95% CI 7·34 to 8·50) to 9·67 (8·34 to 10·17) after one dose, and to 10·17 (8·50 to ≥10·50) after a second dose (table 3). The seroprotection rate was 97% (95% CI 92 to 99) both before and 28 days after one monovalent OPV2 dose, and 98% (89 to 100) after two doses. Seroconversion was observed in 18 (29%) of 62 evaluable participants after one dose of monovalent OPV2, and 11 (38%) of 29 after the second dose.

97 (99%) of 98 in the novel OPV2-c1 groups 1 and 2 and 92 (94%) of 98 in the novel OPV2-c2 groups 3 and 4 were seroprotected before vaccination, and the seroprotection rate was 100% at days 28 (after first dose) and 56 (after second dose) of either novel OPV2 candidate. Median log_2_ titres increased in both groups after one dose, to the ULOQ (10·50) with novel OPV2-c1 and to 10·17 (95% CI 9·67 to ≥10·50) with novel OPV2-c2. A further increase to the ULOQ (10·50) was observed after a second novel OPV2-c2 dose. Measurable seroconversion was observed in 41 (75%) of 55 participants after one dose and 20 (74%) of 27 after two doses of novel OPV-c1. In novel OPV2-c2 vaccinees, seroconversion occurred in 24 (51%) of 47 participants after first dose and 15 (58%) of 26 after the second dose.

At baseline, 42 (89%) of 47 IPV-vaccinated participants were seropositive, increasing to 100% in both novel OPV2 groups after one dose and with median titres greater than the ULOQ. Seroconversion rates were 100% for novel OPV2-c1 and 92% for novel OPV2-c2 (table 3). Although no changes of seroprotection rate or median titre were observed in most placebo recipients, one initially seropositive placebo recipient seroconverted after the second injection.

Viral shedding rates after monovalent OPV2 or novel OPV2 candidates were lower in OPV-vaccinated than in IPV-vaccinated participants, illustrating the induction of intestinal immunity by OPV (table 4). PCR-positive stools were obtained from 15 (15%) of 100 monovalent OPV2 recipients after the first dose. In OPV-vaccinated recipients, 31 (31%) of 100 after the first dose of novel OPV-c1 and 20 (20%) of 100 after novel OPV-c2 had PCR-positive stools. Peak rates of shedding were observed at day 8 after monovalent OPV2, day 7 after novel OPV2-c1, and day 8 after novel OPV2-c2. All assessed participants had stopped shedding poliovirus by day 28 after receiving monovalent OPV2 (n=66) or novel OPV-c1 (n=64), and only one of 66 novel OPV-c2 recipients was still shedding at this timepoint.

**Table 4:** Poliovirus shedding in all vaccinated participants after dose 1 and in the per-protocol population after dose 2

		**Monovalent OPV2 control study**	**Novel OPV2 study—OPV vaccinated**		**Novel OPV2 study—IPV vaccinated**		
		Monovalent OPV2, groups 1 and 2	Novel OPV2-c1, groups 1 and 2	Novel OPV2-c2, groups 3 and 4	Novel OPV2-c1, group 5	Novel OPV2-c2, group 6	Placebo, group 7
**After dose 1**							
N		100	100	100	17	16	17
PCR positive, n (%; 95% CI)		15 (15%; 9–24)	31 (31%; 22–41)	20 (20%; 13–29)	15 (88%; 64–99)	14 (88%; 62–98)	1 (6%; 0–29)
Participants with SIE, n (%)		58 (58%)	94 (94%)	89 (89%)	15 (88%)	15 (94%)	14 (82%)
	Median SIE (95% CI)	0 (0–0)	0 (0–0)	0 (0–0)	1·10 (0·35–2·56)	0·92 (0–1·04)	0 (0–0)
Shedders*, n		11	21	12	12	9	0
	Median SIE in shedders (95% CI)	0·94 (0·92–1·21)	1·07 (0·94–1·41)	0·98 (0·75–1·36)	1·27 (0·86–2·79)	1·03 (0·92–3·44)	NC
**After dose 2**							
N†		50	49	49	17	15	16
PCR positive, n (%; 95% CI)		3 (6%; 1–17)	9 (18%; 9–32)	2 (4%; 1–14)	6 (35%; 14–62)	1 (7%; 0–32)	0 (0%; 0–21)
Participants with SIE, n (%)		27 (54%)	42 (88%)	47 (96%)	13 (77%)	14 (93%)	12 (75%)
	Median SIE (95% CI)	0 (0–0)	0 (0–0)	0 (0–0)	0 (0–0)	0 (0–0)	0 (0–0)
Shedders*, n		1	6	1	1	0	0
	Median SIE in shedders (95% CI)	0·95 (NC)	1·09 (0·92–1·76)	0·92 (NC)	1·05 (NC)	NC	NC

CIs obtained via the percentile bootstrap method. All titres in stool samples with negative shedding results are set to zero; lower limit of quantitation (2·75 log_10_) is used as observed, where necessary. SIE was calculated as the arithmetic mean of log_10_ CCID_50_ per g from days 7, 14, 21, or 28 after dose 1, and days 35, 42, 49, and 56 after dose 2. Medians among shedders were calculated by excluding participants who were PCR negative for shedding at all the respective timepoints. c1=candidate 1. c2=candidate 2. CCID_50_=50% cell culture infective dose. IPV=inactivated poliovirus vaccine. NC=not calculated. OPV=oral poliovirus vaccine. OPV2=type 2 OPV. SIE=shedding index estimate.

* Shedders are participants with non-missing endpoint and with at least one positive result at one of the timepoints used for the respective endpoint.

† Only includes two-dose groups.

In IPV-vaccinated participants, shedding was observed in 15 (88%) of 17 novel OPV2-c1 recipients and 14 (88%) of 16 novel OPV2-c2 recipients. Shedding was effectively finished by day 28 in IPV-vaccinated participants, when one of ten novel OPV-c1 recipients still had a PCR-positive stool, and none of 12 tested in the novel OPV-c2 group were positive. One placebo recipient was found to shed a very low titre of poliovirus in one stool sample collected on day 8. This participant did not display any serological indication of exposure and, although we have no explanation for this observation, it was potentially a case of contamination of the stool sample at the vaccination centre or the laboratory.

After the second dose, numbers of vaccine recipients shedding and the magnitude of viral excretion (CCID_50_) were lower than after the first dose, and similar across groups, including IPV-vaccinated groups (table 4), showing that one dose of either novel OPV2 candidate had induced intestinal immunity.

In OPV-vaccinated participants, genetic stability was assessed in two exploratory endpoint stool samples obtained 5 days after monovalent OPV2, nine samples from days 4–10 after novel OPV2-c1, and five obtained 5–7 days after novel OPV2-c2 (appendix p 6). No variants were observed at the main sites for loss of attenuation, nucleotide 481 or VP1-aa143, or in any other regions of the genome in the monovalent OPV2 samples. In novel OPV2-c1 samples we did not observe any mutations in the relocated cis-acting replication element, including at nucleotides 123 and 179 or at domain IV nt.398 (nucleotide 459 in novel OPV2-c1). No variants consistent with reversion in domain V (nucleotides 468–535), the main determinant for restoration of virulence after monovalent OPV2 administration in humans, or in the Rec1 or Hifi modification locations of the 3D polymerase were observed. Reversion of an unprotected secondary attenuation site, VP1-aa143, was observed in one sample from day 7 but not in samples from days 8, 9, and 10. In novel OPV2-c2 samples, no mutations were observed in domain IV (U398C, equivalent to U459C in novel OPV2 candidate 1) or in domain V, nor any reversions of VP1-aa143 or the modified CpG sites in the P1 region. Of the eight evaluable samples from IPV-vaccinated novel OPV2-c1 recipients, no reverting variants were detected in domain V whereas two day-21 samples showed partial reversion at VP1–143 (appendix p 7). Variants were observed in cis-acting replication element 5 at positions 123/179. In five evaluable samples from IPV-vaccinated novel OPV2-c2 recipients, no reverting variants were observed in domain V but two samples (day 8 and day 9) showed partial reversion at VP1–143.

## Discussion

These two interlinked studies done 2 years apart—a prospective, historical control study using Sabin monovalent OPV2 and the later study with two novel OPV2 candidate vaccines—were designed to compare the novel candidates with monovalent OPV2 in terms of safety and immunogenicity, with exploratory assessments of viral shedding and enhanced genetic stability. We observed that all vaccines were safe and well tolerated, with no serious adverse events or withdrawals definitely related to vaccination. Most solicited systemic adverse events were reported as mild or moderate and transient, with similar reactogenicity profiles for all groups who received monovalent OPV2 or the two novel OPV2 candidates.

Observations of increased creatine phosphokinase and liver enzymes in some participants in the phase 1 study of these novel OPV2 candidates^9^ led to inclusion of additional parameters in the protocol of the novel OPV2 study that had not been included in the monovalent OPV2 study. However, the original suspicion that this was due to excessive exercise by the affected participants living in containment appears to be confirmed, as grade 3 or 4 increases were rare and no consistent changes were observed in this larger novel OPV2 study.

Within the constraints of high baseline immunity, neither novel OPV2 candidate appeared to be inferior immunologically to the monovalent OPV2 vaccine. Although fewer previous vaccinations were registered for the novel OPV2 vaccination study, coverage with four vaccinations is high in Belgium and documented numbers were influenced by availability of vaccination cards. Both novel OPV2 candidates were also immunogenic in IPV-immunised adults, with 100% seroprotection rates after one dose, as previously shown in the phase 1 study.^9^

Both novel OPV2 candidates and monovalent OPV2 were shed in stools at a similar rate in OPV-vaccinated participants. Shedding was higher in IPV-vaccinated participants, which is expected because, unlike OPV, IPV induces little to no primary intestinal immunity.^14^ Peak rates of shedding were observed within 10 days of vaccination and virtually all participants had stopped shedding within the 28-day follow-up period. For the novel OPV2 candidates, the sequencing results remain promising and consistent with the phase 1 study^9^ with no reverting modifications of the genetically stabilised domain V detected in any samples from any cohorts, while Sabin-2 reversion in domain V is common at day 7 after vaccination and beyond.^12^, ^15^ More detailed analysis of the genetic variations together with ongoing analyses of the neurovirulence of the shed virus will be reported subsequently.

With increasing numbers of circulating vaccine-derived poliovirus outbreaks globally, the WHO–Global Polio Eradication Initiative strategy to interrupt transmission relies on the development of new vaccines with more genetically stable poliovirus strains like those described here.^5^ With reports of outbreaks due to types 1 and 3, development of similar novel OPV candidates for types 1 and 3 has already been initiated and a phase 1 study with these new candidate vaccines is scheduled to start in early 2021 (NCT04529538).

The main limitation of this investigation was the necessity to do two separate studies. Global withdrawal of Sabin OPV2 in 2016 before novel OPV2 lots became available made direct contemporaneous comparison of monovalent OPV2 and novel OPV2 candidates impossible, necessitating the historical study for monovalent OPV2 baseline data. To enable comparisons between studies, both protocols were designed to be as similar as possible using volunteers from the same population in Belgium. Although essentially open label for safety because monovalent OPV2 was studied first, immunogenicity analyses were done simultaneously in a masked manner in the same laboratory to minimise potential bias. We assessed novel OPV2 shedding in participants with different background polio vaccination histories because exclusively IPV-vaccinated participants have low or no intestinal immunity, unlike OPV vaccinees. As well as circulating reverted viruses, other rare consequences of OPV use are cases of vaccine-associated paralytic poliomyelitis, occurring in vaccinees or their contacts at a rate of about four cases per million births.^16^ Clinical studies, including this one, are too small to detect such a phenomenon so it is speculative whether the improved genetic stability of novel OPV2 will have an effect on rates of vaccine-associated paralytic poliomyelitis. Another limitation is that this was done in fully vaccinated adults, whereas the most likely recipients of novel OPV2 will be children and infants, who might be unvaccinated or incompletely immunised. For that reason, following initial safety assessments by the data and safety monitoring board of the present adult novel OPV2 study, a study of both novel OPV2 candidates (with a historical monovalent OPV2 control study) was done in Panama in children and bivalent OPV-immunised or IPV-immunised infants to simulate the situation with minimal intestinal immunity against type 2 virus in the post-OPV2 withdrawal era. Results of these studies are presented in an accompanying paper.^10^

In our studies, both novel OPV2 candidates appeared to be as safe, well tolerated, and immunogenic as monovalent OPV2, with similar profiles of viral shedding. Further study is underway to confirm the objective of mitigating reacquisition of neurovirulence by these novel OPV2 vaccines, but the data thus far suggests that the goal of developing more genetically stable, attenuated OPV2s with no effect on the immunogenicity has been achieved.

## Data sharing

Data for this study will be made available to others in the scientific community upon request after the ongoing phase 3 study of the selected vaccine candidate has been completed and the scientific data from the development of both candidates have been fully published. Standard criteria for making data available for valid research projects will be used, following application by suitably qualified researchers. For data access, please contact the Gates Foundation (openaccess@gatesfoundation.org).
