# Supplementary material for: Safety and immunogenicity of two novel type 2 oral poliovirus vaccine candidates compared with a monovalent type 2 oral poliovirus vaccine in healthy adults: two clinical trials
Source: Lancet. 2021 Jan 2;397(10268):39–50. doi: 10.1016/S0140-6736(20)32541-1 (PMC7811203; doi:10.1016/S0140-6736(20)32541-1)
Supplement: Supplementary appendix [file mmc1.pdf]

THE LANCET

Supplementary appendix

This appendix formed part of the original submission and has been peer reviewed. We post it as supplied by the authors.

Supplement to: De Coster I, Leroux-Roels I, S Bandyopadhyay A, et al. Safety and immunogenicity of two novel type 2 oral poliovirus vaccine candidates compared with a monovalent type 2 oral poliovirus vaccine in healthy adults: two clinical trials. *Lancet* 2020; published online Dec 9. http://dx.doi.org/10.1016/S0140-6736(20)32541-1.

De Coster et al

.

Appendix material.

Table 1. Solicited Adverse Events after first dose page 2

Table 2. Solicited Adverse Events after second dose page 3

Table 3. Clinically-relevant laboratory abnormalities page 4

Table 4. Primary immunogenicity objective data page 5

Table 5. Frequency of genetic variants – OPV-vaccinated adults page 6

Table 6. Frequency of genetic variants – IPV-vaccinated adults page 7

Table 1. Summary of Solicited Adverse Events (AEs) after the first dose – TVP.

		Historical control study	nOPV2 study OPV-vaccinated		nOPV2 study IPV-vaccinated		
**Category**	**Severity***	**Groups 1 & 2**	**Groups 1 & 2**	**Groups 3 & 4**	**Group 5**	**Group 6**	**Group 7**
	**Vaccine**	mOPV2	nOPV2-c1	nOPV2-c2	nOPV2-c1	nOPV2-c2	Placebo
				**n subjects (%)**			
**Solicited AEs**		N = 100	N = 100	N = 100	N = 17	N = 16	N = 17
	Any	62 (62)	71 (71)	74 (74)	16 (94)	13 (81)	15 (88)
	Severe	*4 (4)*	*3 (3)*	*2 (2)*	*1 (6)*	*0*	*2 (12)*
**Abdominal pain**	Any	19 (19)	21 (21)	20 (20)	8 (47)	3 (19)	8 (47)
	Severe	*0*	*0 (0)*	*0*	*0*	*0*	*0*
**Anaesthesia**	Any	1 (1)	2 (2)	0	-	-	-
	Severe	*0*	*0*	*0*			
**Arthralgia**	Any	4 (4)	9 (9)	4 (4)	2 (12)	0	0
	Severe	*0*	*0*	*0*	*0*	*0*	*0*
**Diarrhoea**	Any	20 (20)	22 (22)	24 (24)	9 (53)	3 (19)	5 (29)
	Severe	*0*	*0*	*0*	*0*	*0*	*0*
**Fatigue**	Any	35 (35)	37 (37)	35 (35)	10 (59)	9 (56)	9 (53)
	Severe	*0*	*0*	*1 (0)*	*0*	*0*	*2 (12)*
**Fever**	Any	4 (4)	3 (3)	2 (2)	1 (6)	3 (19)	1 (6)
	Severe	*0*	*0*	*0*	*0*	*0*	*0*
**Headache**	Any	28 (28)	39 (39)	48 (48)	12 (71)	9 (56)	9 (53)
	Severe	*3 (3)*	*2 (2)*	*1 (0)*	*1 (6)*	*0*	*1 (6)*
**Myalgia**	Any	12 (12)	16 (16)	12 (12)	3 (18)	3 (19)	2 (12)
	Severe	*1 (1)*	*1 (1)*	*0*	*0*	*0*	*0*
**Nausea**	Any	8 (8)	18 (18)	9 (9)	4 (24)	3 (19)	5 (29)
	Severe	*0*	*0*	*0*	*0*	*0*	*0*
**Paresthesia**	Any	6 (6)	5 (5)	3 (3)	3 (18)	3 (19)	1 (6)
	Severe	*1 (1)*	*0*	*0*	*0*	*0*	*0*
**Vomiting**	Any	2 (2)	0	2 (2)	0	2 (13)	0
	Severe	*0*	*0*	*0*	*0*	*0*	*0*

Table 2. Summary of Solicited Adverse Events (AEs) after the second dose – TVP.

		Historical control study	nOPV2 study OPV-vaccinated		nOPV2 study IPV-vaccinated		
**Category**	**Severity***	**Group 2**	**Group 2**	**Group 4**	**Group 6**	**Group 5**	**Group 7**
	**Vaccine**	mOPV2	nOPV2-c1	nOPV2-c2	nOPV2-c2	nOPV2-c1	Placebo
		**n subjects (%)**					
**Solicited AEs**		N = 50	N = 49	N = 49	N = 17	N = 15	N = 16
	Any	18 (38)	26 (53)	21 (43)	11 (65)	9 (60)	12 (75)
	Severe	*1 (2)*	*0*	*1 (2)*	*0*	*1 (7)*	*0*
**Abdominal pain**	Any	3 (6)	8 (16)	2 (4)	5 (29)	4 (27)	4 (25)
	Severe	*0*	*0 (0)*	*1 (1)*	*0*	*0*	*0*
**Anaesthesia**	Any	1 (2)	0	0	-	-	-
	Severe	*0*	*0*	*0*			
**Arthralgia**	Any	1 (2)	3 (6)	2 (4)	0	1 (7)	0
	Severe	*0*	*0*	*0*	*0*	*0*	*0*
**Diarrhoea**	Any	6 (12)	9 (18)	4 (8)	7 (41)	2 (13)	3 (19)
	Severe	*0*	*0*	*0*	*0*	*0*	*0*
**Fatigue**	Any	9 (18)	14 (29)	9 (18)	6 (35)	7 (47)	10 (63)
	Severe	*1 (2)*	*0*	*0*	*0*	*1(7)*	*0*
**Fever**	Any	0	2 (4)	1 (2)	0	1 (7)	0
	Severe	*0*	*0*	*0*	*0*	*0*	*0*
**Headache**	Any	8 (16)	15 (31)	13 (27)	7 (41)	5 (33)	6 (38)
	Severe	*0*	*0*	*0*	*0*	*0*	*0*
**Myalgia**	Any	4 (8)	2 (4)	1 (2)	0	4 (27)	4 (25)
	Severe	*0*	*0*	*0*	*0*	*0*	*0*
**Nausea**	Any	2 (4)	4 (8)	1 (2)	1 (6)	1 (7)	4 (25)
	Severe	*0*	*0*	*0*	*0*	*0*	*0*
**Paresthesia**	Any	2 (4)	0	0	1 (6)	1 (7)	1 (6)
	Severe	*0*	*0*	*0*	*0*	*0*	*0*
**Vomiting**	Any	1 (2)	0	0	0	0	0
	Severe	*0*	*0*	*0*	*0*	*0*	*0*

Table 3. Clinically relevant (Grade 3 or 4) laboratory abnormalities up to 28 days after vaccination in TVP of the two studies.

		Historical control study		nOPV2 study						
				OPV-vaccinated				IPV-vaccinated		
	**Group**	Group 1	Group 2	Group 1	Group 2	Group 3	Group 4	Group 5	Group 6	Group 7
	**Vaccine**	mOPV2	mOPV2	nOPV2-c1		nOPV2-c2		nOPV2-c1	nOPV2-c2	Placebo
**Laboratory assessment**	N =	100	50	100	50	100	50	17	16	17
**Any clinically relevant change**		21 (21)	18 (36)	28 (28)	18 (36)	30 (30)	15 (30)	4 (24)	6 (38)	9 (53)
**Any Grade 3 or 4 anomaly**										
**Albumin**	n (%)	nd	nd	0 (0)	0 (0)	0 (0)	0 (0)	0 (0)	0 (0)	0 (0)
**APTT**		0 (0)	0 (0)	0 (0)	0 (0)	0 (0)	0 (0)	0 (0)	0 (0)	0 (0)
**ALT**		0 (0)	0 (0)	0 (0)	0 (0)	0 (0)	0 (0)	0 (0)	0 (0)	0 (0)
**AST**		0 (0)	0 (0)	0 (0)	0 (0)	0 (0)	0 (0)	0 (0)	0 (0)	0 (0)
**CPK**		nd	nd	0 (0)	0 (0)	2 (2)	0 (0)	2 (12)	0 (0)	3 (18)
**Creatinine**		0 (0)	0 (0)	0 (0)	0 (0)	0 (0)	0 (0)	0 (0)	0 (0)	0 (0)
**Fibrinogen**		0 (0)	0 (0)	0 (0)	0 (0)	0 (0)	0 (0)	0 (0)	0 (0)	0 (0)
**GGT**		nd	nd	0 (0)	0 (0)	0 (0)	0 (0)	0 (0)	0 (0)	0 (0)
**Glucose**		0 (0)	0 (0)	1 (1)	0 (0)	0 (0)	0 (0)	0 (0)	0 (0)	0 (0)
**Haemoglobin**		0 (0)	0 (0)	0 (0)	0 (0)	0 (0)	0 (0)	0 (0)	0 (0)	0 (0)
**Lymphocytes**		0 (0)	0 (0)	0 (0)	0 (0)	0 (0)	0 (0)	0 (0)	0 (0)	0 (0)
**Neutrophils**		0 (0)	1 (2)	1 (1)	0 (0)	0 (0)	0 (0)	0 (0)	0 (0)	0 (0)
**Phosphate**		0 (0)	0 (0)	0 (0)	0 (0)	1 (1)	3 (6)	0 (0)	0 (0)	0 (0)
**Platelets**		0 (0)	0 (0)	0 (0)	0 (0)	0 (0)	0 (0)	0 (0)	0 (0)	0 (0)
**Potassium**		0 (0)	1 (2)	0 (0)	0 (0)	0 (0)	0 (0)	1 (6)	1 (6)	1 (6)
**Total bilirubin**		0 (0)	1 (2)	0 (0)	0 (0)	0 (0)	0 (0)	0 (0)	0 (0)	0 (0)
**White blood cell count**		0 (0)	0 (0)	0 (0)	0 (0)	0 (0)	0 (0)	0 (0)	0 (0)	0 (0)

nd = not determined. APTT = activated partial thromboplastin time; ALT = alanine aminotransferase; AST = aspartate aminotransferase; CPK = creatine phosphokinase; GGT = gamma-glutamyl transferase.

Table 4. Primary immunogenicity objective: seroprotection rate 28 days after one dose of mOPV2 or nOPV2 candidates in the PPP

		Historical control study	nOPV2 study - OPV vaccinated		ΔSPR, % (95% CI)	
		**mOPV2** *Groups 1 and 2*	**nOPV2-c1** *Groups 1 and 2*	**nOPV2-c2** *Groups 3 and 4*	**nOPV2-c1 – mOPV2**	**nOPV2-c2– mOPV2**
**Seroprotection rates**[a]	**(n/N)** % (95% CI)					
Day 0		**97 / 100** 97 (92–99)	**97 / 98** 99 (94–100)	**92 / 98** 94 (87–98)	2·0 (-2·9–7·6)	-3·1 (-10·1–3·2)
Day 28		**98 / 100** 98 (93–100)	**96 / 96** 100 (96–100)	**98 / 98** 100 (96–100)	2·0 (-1·9–7·0)	2·0 (-1·8–7·0)
**Median neutralising titres**[b]	(Q1–Q3)					
Day 0		**228** (144–362)	**324** (228–455)	**455** (256–724)		
Day 28		**815** (324–1152)	**≥ 1448** (≥1448– ≥1448)	**1152** (815– ≥1448)		

a: Seroprotection rate based on type 2 neutralising titres, calculated as n/N x 100.

b: Interquartile statistics calculated on log_2-transformed type 2 neutralising titres back transformed for this table.

Table 5. Frequency of genetic variants at known attenuation sites and modified regions of the candidates in OPV-vaccinated adults.

	Genome unit (nucleotides)	**cre5 (121-181)**	**Dom IV 459**	**S15 Dom V (529-596)**	**VP1-143 (2969-2971)**	**2C cre KO (4508-4560)**
				**nOPV2-c1 in OPV-vaccinated adults**		
	Variant observed[2]			C547U	A2969G (I143V)	U4540C
EES Day[1]	D4 (SI)	n.d.	n.d.	n.d.	n.d.	n.d.
	D6 (SSI)	n.d.	n.d.	n.d.	n.d.	0, 0, 0.01
	D6 (SSI)	n.d.	n.d.	n.d.	n.d.	n.d.
	D7 (SSI)	n.d.	n.d.	n.d.	0·56, 0·52, 0·97	n.d.
	D7(SSI)	n.d.	n.d.	n.d.	n.d.	0·02, 0·03, 0
	D8 (SSI)	n.d.	n.d.	n.d.	n.d.	n.d.
	D9 (SS)	n.d.	n.d.	n.d.	n.d.	n.d.
	D10 (S)	n.d.	n.d.	n.d.	n.d.	n.d.
	D10 (SS)	n.d.	n.d.	0·21, 0	n.d.	n.d.
				**nOPV2-c2 in OPV-vaccinated adults**		
				**No variations or mutations were observed in EES from Day 5 to Day 7**		

Table 6. Frequency of genetic variants at known attenuation sites and modified regions of the candidates in IPV-vaccinated adults.

					**nOPV2-c1 in IPV-vaccinated adults**			
	Variant observed[2]	U123C/G179A	U459C		VP1 A2969G (I143V)	VP1 U2970C (I143T)	VP1 U2970G (I143S)	C4519U
EES Day[1]	D3 (SSI)	n.d.	n.d.	n.d.	n.d.	n.d.	n.d.	0, 0.03, 0.02
	D6 (SI)	n.d.	n.d.	n.d.	n.d.	n.d.	n.d.	n.d.
	D8 (I)	n.d.	n.d.	n.d.	0.04	n.d.	n.d.	n.d.
	D8 (SSI)	1·00, 0·97, 1·00	n.d.	n.d.	n.d.	n.d.	n.d.	n.d.
	D9 (SI)	0, 0·03	n.d.	n.d.	n.d.	n.d.	n.d.	n.d.
	D9 (SSI)	0·31, 0·05, 0·53	0·05, 0, 0·10	n.d.	n.d.	n.d.	n.d.	n.d.
	D21 (SSI)	0·96, 0·96, 0·55	0, 0·01, 0	n.d.	0·53, 0·52, 0·56	0·03, 0·04, 0·07	0·09, 0·12, 0·03	n.d.
	D21 (SI)	0·07, 0·38	0·93, 0·62	n.d.	n.d.	0·10, 0·38	n.d.	n.d.
					**nOPV2-c2 in IPV-vaccinated adults**			
	Variant observed[2]		Dom IV 398	S15domV (468-535)	U2909A (I143N)	U2909C (I143T)		
EES Day[1]	D4 (SSI)		n.d.	n.d.	n.d.	n.d.		
	D8 (SI)		n.d.	n.d.	0·08, 0·01	0, 0·15		
	D9 (SS)		n.d.	n.d.	n.d.	n.d.		
	D9 (SSI)		n.d.	n.d.	0·16, 0·25, 0·02	0, 0·02, 0		
	D10 (SSI)		n.d.	n.d.	n.d.	n.d.		

[1] EES day shown with stool 1, stool 2 and cell culture isolate (SSI), if present;

[2] Associated amino acid change indicated;

n.d. = Variant not detected in stool or isolate

Variants at ≥1% (0.01) are reported; value of 0 presented within a sample set means not detected (<1%)
